# Deformability of Heterogeneous Red Blood Cells in Aging and Related Pathologies

**DOI:** 10.14336/AD.2024.0526

**Published:** 2025-06-19

**Authors:** Dmitry S. Prudinnik, Aigul Kussanova, Ivan A. Vorobjev, Alexander Tikhonov, Fazly I. Ataullakhanov, Natasha S. Barteneva

**Affiliations:** ^1^Department of Biology, School of Sciences and Humanities, Nazarbayev University, Astana 010000, Kazakhstan.; ^2^Department of Physiology, Perelman School of Medicine, University of Pennsylvania, Philadelphia, PA 19104, USA.

**Keywords:** Aging, Cellular deformability, Neurodegenerative diseases, Erythrocytes, Yoda1, PIEZO1, Alzheimer’s Disease, Parkinson Disease, Imaging flow cytometry

## Abstract

Aging is interrelated with changes in red blood cell parameters and functionality. In this article, we focus on red blood cells (RBCs) and provide a review of the known changes associated with the characterization of RBC deformability in aging and related pathologies. The biophysical parameters complement the commonly used biochemical parameters and may contribute to a better understanding of the aging process. The power of the deformability measurement approach is well established in clinical settings. Measuring RBCs' deformability has the advantage of relative simplicity, and it reflects the complex effects developing in erythrocytes during aging. However, aging and related pathological conditions also promote heterogeneity of RBC features and have a certain impact on the variance in erythrocyte cell properties. The possible applications of deformability as an early biophysical biomarker of pathological states are discussed, and modulating PIEZO1 as a therapeutic target is suggested. The changes in RBCs' shape can serve as a proxy for deformability evaluation, leveraging single-cell analysis with imaging flow cytometry and artificial intelligence algorithms. The characterization of biophysical parameters of RBCs is in progress in humans and will provide a better understanding of the complex dynamics of aging.

## 1. Introduction

Red blood cells (RBCs) comprise an overwhelming majority (>98%) of all blood cells and 40% of blood by volume. Their main function is to deliver oxygen to tissues and eliminate carbon dioxide. Erythrocyte counts and hemoglobin (Hb) levels tend to decrease in the elderly [1], which may lead to reduced oxygen delivery. To carry out a gas exchange with the tissues, an erythrocyte with a diameter of 6-8 µm and 2 µm thickness must be able to pass the microcapillaries with a diameter of 2-5 µm. This is possible due to the extreme deformability of RBC. The deformability of RBCs is directly linked to RBC membrane rigidity and elasticity [2-5] and is an essential parameter of blood microviscosity, which determines usefulness and viability of mammalian erythrocytes in an organism [6]. The erythrocyte deformability changes in older adults, and is affected by various pathological conditions, and can have significant effects on blood flow. The dysregulated erythrocyte function contributes to the pathophysiology of neurodegenerative diseases, including Alzheimer's disease (AD). Recent research aims to identify potential mechanobiological biomarkers, which can be exploited for diagnostics and therapeutic monitoring purposes [7-11].

For a long time, methods to study erythrocyte deformability provided data averaged for large cell populations without considering the heterogeneity of RBCs. Recent advancements in engineering allow for the evaluation of deformability at a single-cell level or even of the local areas of a single cell [12-13]. The combination of micropipette aspiration, optical tweezers, and fluorescent labeling technology has been applied to study erythrocyte deformation at the molecular level. On the other hand, the intention to create a simple, clinically applicable method promotes the development of techniques to evaluate a large number of cells without neglecting a small population of non-deformable cells. This review analyzes RBC deformability in aging and related pathological states. We hypothesize that RBCs’ deformability and related biophysical features encoded in cells can serve as early biomarkers of pathological processes and may shed light on the aging process and the development of chronic disease states in the elderly. Moreover, we discuss the methods to quantify the deformability of RBCs and future perspectives of the field.

## 2. Deformability of RBCs in aging

### 2.1 Mechanical properties of RBCs

**Figure 1.**
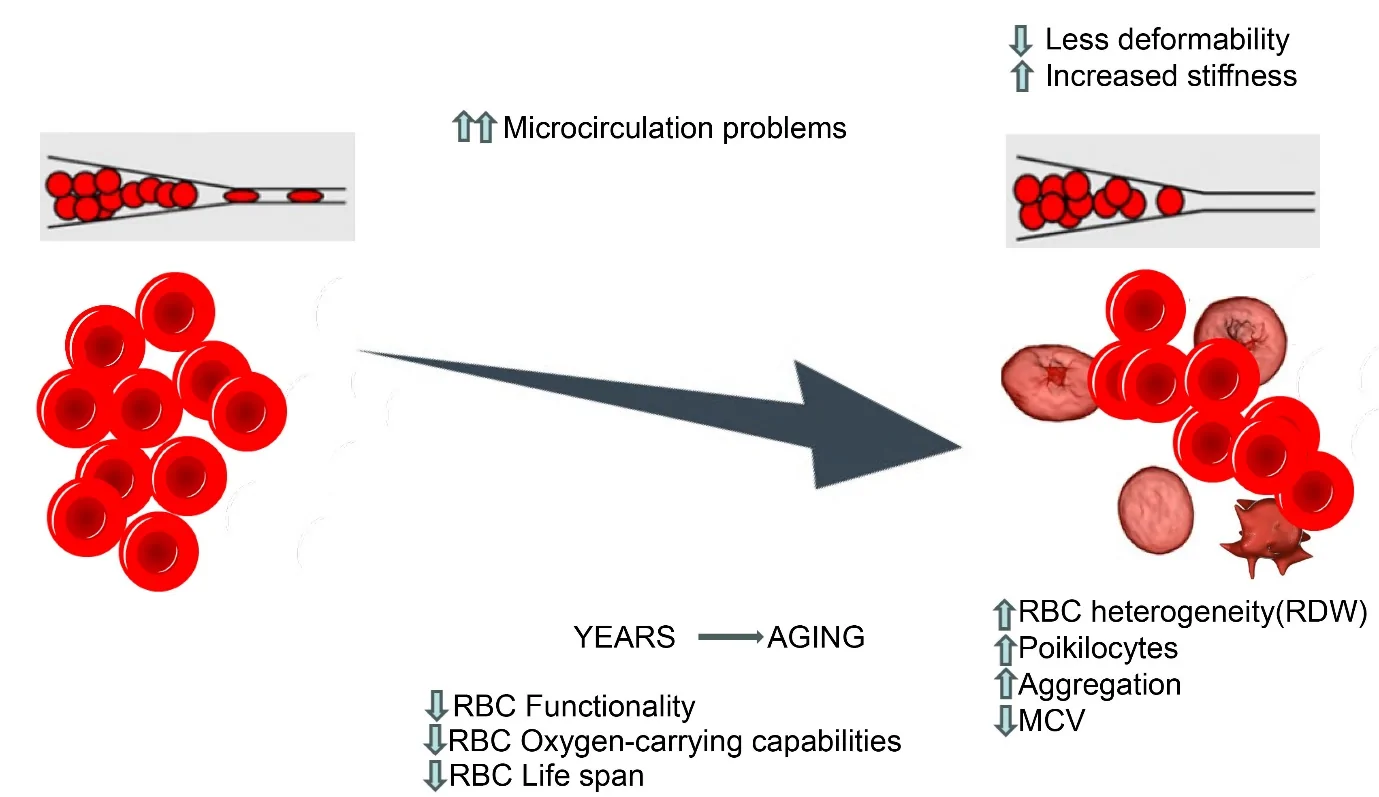
Changes in RBC parameters in aging population.

The mechanobiological properties of erythrocytes, particularly erythrocyte deformability, change in the elderly (Fig. 1). Erythrocyte deformability is mainly influenced by the surface area to volume ratio (S/V), internal viscosity depending on intracellular Hb concentration and its physico-chemical state, and viscoelastic properties of the membrane, which are largely determined by the cytoskeleton - an actin-spectrin network that underlies the inner leaflet of the erythrocyte membrane [14-16]. The systematic fluid dynamic of RBC suspensions flowing in microcapillaries *in vitro* was reported by Tomaiuolo and co-authors [17-19]. Normal RBC has a biconcave discocyte shape, which provides excess surface area over that required to enclose the cell volume in a sphere. The deformation of the erythrocyte membrane occurs without changing the surface area. The erythrocytes change in shape in microvasculature from slipper-like to croissant-like, and various dynamical states have been reported in experimental and numerical simulation studies [20-22].

RBCs are able to undergo extreme deformations allowing them to pass through rigid slits as narrow as 0.28 µm at body temperature [23].

#### 2.1.1 RBCs and PIEZO1

The PIEZO1, a stretch-activated mechanosensitive Ca^2+^ channel in RBC, exhibits the properties of a force-through sensor of curvature [24] and is involved in regulating erythrocyte volume [25], shape change, and clot formation [26]. Patapoutian’s group identified a small family of evolutionary conserved mechanosensitive channels in 2010, which allow calcium and other ions to enter the cell [27-28]. The crucial role of PIEZO1 was first described in erythrocytes; gain-of-function mutations in *PIEZO1* gene have been linked to xerocytosis, a hereditary disease affecting erythrocytes ([29-32], and to lymphatic disorders ([33-35]. Furthermore, PIEZO1 activity is lower in reticulocytes [36], and an increase in PIEZO1 activity delays erythroid maturation [37]. Moreover, hyperglycemia activates *PIEZO1* transcription in mature RBCs [38], and elevated PIEZO1 activity in RBCs, platelets, and neutrophils in patients with type 2 diabetes triggers prothrombotic cellular responses [38]. PIEZO1 is involved in regulating key biological functions, such as cell volume and shape, cell migration, differentiation, and cell proliferation, leading to the accumulation of cells in G0/G1 of the cell cycle [37, 39-41].

The PIEZO1 is selectively activated by a synthetic small molecule, Yoda1, identified by high-throughput screening [42] and their analogs [42, 43]. However, the nature and extent of Yoda1 effects on PIEZO1 remain unclear. Activation of PIEZO1 orchestrates amyloid beta clearance [44] and improves neurocognitive functions in craniostosis murine models [45]. It was also found that PIEZO1 is a key regulator of lymphatic valve formation [46-48], and lymphatic valves are essential for the proper flow of lymph throughout the body. Changes in meningeal lymphatic function slow the paravascular influx of macromolecules into the brain and induce cognitive impairment in mouse models [49]. Meningeal lymphatic dysfunction in transgenic mouse models of Alzheimer’s disease aggravates meningeal and parenchymal amyloid beta accumulation [49]. Meningeal lymphatic vessels (mLVs) have recently been demonstrated to be an important clearance pathway in the brain and are involved in neurodegenerative diseases pathogenesis [50-52]. The aging process induces changes in the structure and function of systemic and intracranial lymphatic networks [53]. Augmentation of mLVs and glymphatic function might be a promising therapeutic target for delaying and preventing age-associated neurological diseases. Recently, Matrongolo and co-authors (2023) have found using craniosynostosis murine model (exacerbates amyloid pathology and plaque buildup) and aging mice that Yoda1 treatment reduced CSF flow and turnover, improves lymphatic networks, drainage, and brain-CSF perfusion [54]. Furthermore, Choi and co-authors have found using murine models that activate PIEZO1 through transgenic overexpression or treatment with the chemical agonist Yoda1 improves lymphatic absorption and transport and increases CSF outflow [55]. Importantly, these works open a road for potential therapeutic treatments.

**Figure 2.**
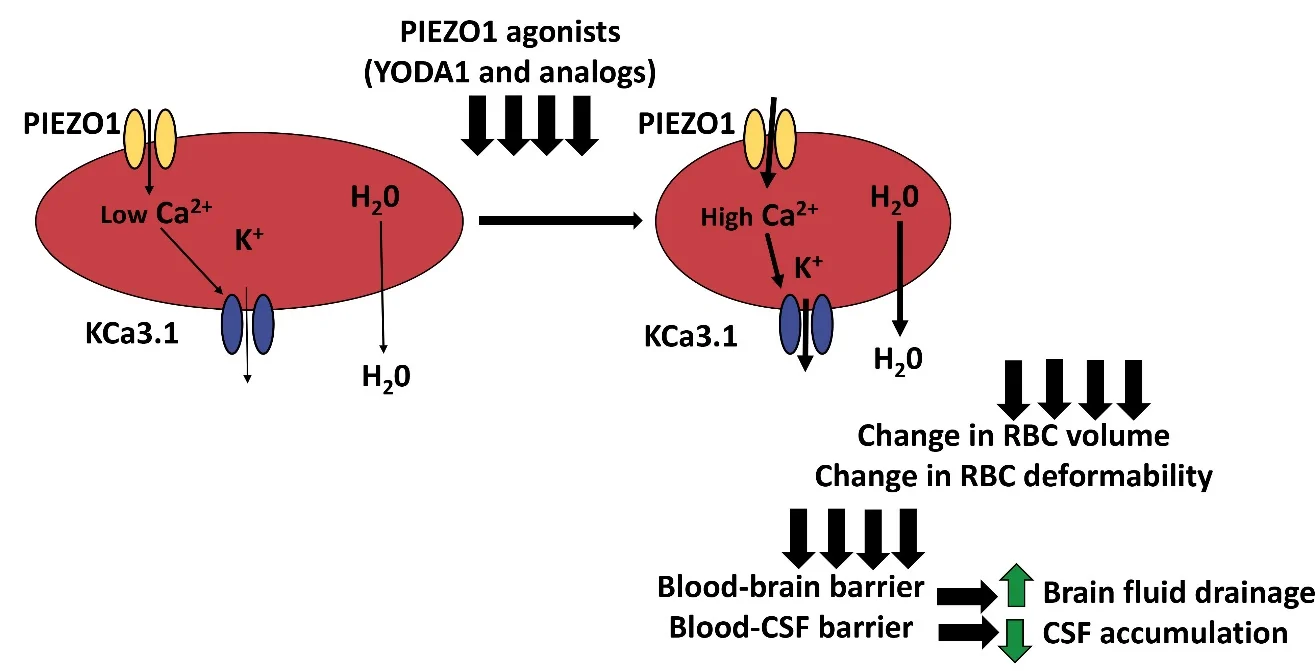
**PIEZO1 activation by agonists (Yoda1) in animal experiments regulates CSF outflow [45, 47] and orchestrates amyloid beta clearance [44]**. We hypothesize that the treatment with Yoda 1 and small molecule agonists may lead to activation of PIEZO1 and change in cell deformability in human erythrocytes.

Since there are at least two major phenotypes involving *PIEZO1* gene that are related to RBCs and the lymphoid system [35], the effect of PIEZO1 on RBCs in aging should not be overlooked. The process of human aging is intricately linked with a decrease in RBC deformability [56-59]. However, the exact details of PIEZO1 effects on RBCs are still under development. The short transit times of RBCs through narrow slits (<100 milliseconds) making not clear how the potential activation of PIEZO1 and Gardos channels modulate RBC volume during this transition [23] (Fig. 2). Under normal physiological conditions, the deformability of erythrocytes enables adequate blood flow.

The most pronounced changes in deformability were observed under low shear stresses and hyperosmotic conditions [60]. It has been observed that a decrease in RBC deformability leads to a significant increase in microvascular flow resistance and blood viscosity [61]. RBC deformability is a key determinant of blood flow in microcirculation [62]. Even a slight decrease in RBC deformability can impact blood flow, tissue perfusion, oxygenation [63-65], and the integrity of the blood-brain barrier (BBB) [66]. Pharmacological interventions using agents that disturb the RBC membrane organization can change RBC deformability [67]. For instance, sodium orthovanadate and metavanadate can reduce erythrocyte deformability [68-69], and vanadate can inhibit the stomatocytic shape change induced by amphipathic cationic drugs; however, it does not modify changes induced by low pH [70]. Findings from pharmacological interventions and labeling studies suggest the involvement of band 3 protein, associated glycolytic proteins, and membrane proteins. However, detailed signal transduction pathways involved in the RBC shape change are not yet fully understood [71]. Nevertheless, there have been published correlations between the levels of RBC membrane proteins (including CD44, CD47, glycophorins, ezrin, and others) and cell deformability (expressed by the average elongation ratio) [72].

### 2.2 Heterogeneity of RBCs population

RBCs are a heterogeneous population of cells of different ages. The average life span of RBCs in healthy subjects is approximately 115-126 days after they are released from bone marrow [73-74]. Reticulocytes are the erythrocyte progenitors that represent 0.5 to 2.5% of adult blood RBCs and remain in WHO reference values in hematologically healthy elderly subjects (> 60 years) [75]. However, their numbers may decrease in individuals over 80 years old [76]. Reticulocytes have different biomechanical properties compared to mature RBCs, including lower deformability and membrane fluidity, as well as greater viscoelastic modulus from erythrocytes [77-78]. The deformability of RBCs increases by 50-fold during erythropoiesis and has been associated with a release of RBCs from marrow for a long time [79]. During the circulation of RBCs, there are many cyclic changes in shear stresses, oxygen pressure, pH, and oxidative stress. This variable microenvironment leads to the senescence and removal of the RBCs [80]. The average RBC life span, calculated using isotope techniques, is considerably reduced in older animals [81-82] and aged human donors [83]. Senescent RBCs undergo a number of physical and chemical changes, and potential mechanisms for red cell senescence include the generation of reactive oxygen species (ROS), mechanical fatigue [84], and ATP depletion [85]. The heterogeneity of the erythrocyte population can also be attributed to various pathological conditions that disrupt the microenvironment of the erythrocyte or alter the structure and metabolism of the erythrocyte itself. Increased variability in the volume and RBC shape can be caused by excessive vesiculation during impaired erythropoiesis or by excessive fragmentation and destruction. The RBCs heterogeneity expressed as RDW increase (see 5.1) is associated with increased odds of having dementia [86] and associated with leukoaraiosis [87], mild cognitive impairment [88], AD [89], the severity of metabolic syndrome [90-91], and also increased in prehypertension and hypertension [92-95]. Finally, erythrocyte heterogeneity can stem from macroenvironment of the individual, such as changes in exercising, diet, living in the highlands with hypoxic conditions, etc. Furthermore, individual RBCs demonstrate varying abilities to respond to different stresses and stimuli. For instance, some RBCs may be more susceptible to oxidative stress than others [96], and different subpopulations of RBCs may respond differently to shear stress [97]. This phenotype plasticity and heterogeneity contribute to the high variability of deformability in various RBC subpopulations. A comprehensive review of this topic can be found in a publication by Bogdanova and co-authors [98].

## 3. RBCs deformability in aging-related pathologies

Neuroinflammation, oxidative stress, and neurodegeneration are often associated with aging and age-related diseases. Chronic inflammation, pathological protein aggregation, cytoskeletal abnormalities, and altered energy homeostasis were revealed as major factors contributing to the development of neurodegenerative diseases [99-101]. The process of aging is associated with a decline in the functionality and oxygen-carrying capabilities of RBCs [102-104]. However, it is necessary to distinguish between specific changes in erythrocytes due to aging and the effects of diseases that are prevalent in old age.

### 3.1 Metabolic syndrome and type II diabetes

Disorders that do not primarily affect RBCs but decrease their deformability include metabolic disorders such as hypercholesterolemia and diabetes [105-107]. It is known that changes in fatty acid (FA) concentrations in erythrocytes affect lipid fluidity in the membrane and overall erythrocyte deformability [108-109]. Some studies suggest using membrane fluidity as a sensitive marker to distinguish subjects with type 2 diabetes and low cardiovascular risk from those with very high cardiovascular risk [110]. The FA blood profile changes with age, and long-lived individuals have a different profile characterized by high concentrations of monounsaturated FA [111]. Type 2 diabetes is associated with notable changes in the lipid composition of red blood cells, including higher levels of saturated fatty acids and increased content of cholesterol, total sphingolipids, and sphingomyelin [112]. These changes lead to an increase in membrane rigidity due to the higher proportion of membrane cholesterol and the cholesterol to phospholipid ratio [113].

Diabetes is one of the most intensely studied metabolic diseases when it comes to RBC deformability [114-117]. Studies using atomic force microscopy (AFM) and scanning electron microscopy (SEM), have found that in type 2 diabetes patients, RBCs have decreased diameter, height, and surface area, irregular elongated shape, and higher stiffness compared with erythrocytes from healthy subjects [117-119, 5]. The advanced glycation end products (AGEs) were attributed to aging and diabetes of both types [120-121]. The most studied and clinically relevant glycosylated hemoglobin (HbA1c), which is now considered to be a diabetes marker [122-123] and, as demonstrated by some researchers, was associated with impaired erythrocyte deformability [124]. Other suggested defects of RBCs caused by diabetes of both types are glycosylation of membrane and cytoskeletal proteins (such as spectrin, ankyrin, and protein 4.2) [125-127], which increase cell rigidity. Additionally, erythrocytes of patients with type II diabetes have been shown to have increased levels of the indicators of lipid peroxidation, decreased glutathione levels, and membrane -SH group content, i.e., reduced antioxidant activity [128]. The number of the SH-groups on spectrin is considered to be a function of erythrocyte membrane deformation [129].

### 3.2 Hypertension and cardiovascular pathologies

Hypertension, stroke, and cardiovascular diseases are linked to functional and morphological abnormalities of RBCs. Blood pressure levels and fluctuations had a greater impact on RBCs than body mass index and hemoglobin glycosylation. Altered mechanical properties of erythrocytes, decreased membrane fluidity [130], elongation index (EI) [131], and decrease in erythrocyte deformability have been linked to hypertension [132-134], particularly with the severity of hypertension [135], and coronary risk in the elderly [136]. A slight decrease in deformability was observed in patients with hypertension using a nickel mesh filtration technique [137].

Hypertension was found to be associated with decreased activity of antioxidant enzymes, alterations in lipid composition [138], changes in erythrocyte membrane transporters [138], increased levels of lipid peroxidation [131], Na/K-ATPase activity [134], and sulfhydryl groups in membrane proteins. Erythrocyte membranes from individuals with high hypertension had low cholesterol content, increased phosphatidylcholine, phosphatidylamine, and phospatidylserine levels, increased membrane fragility and fluidity, and were more prone to eryptosis. Additionally, the activity of Na/K-ATPase and Ca^2+^ATPase decreases with age, along with decreased antioxidant activity as assessed by the ferric-reducing ability of plasma [140]. The abnormal response of erythrocytes to oxidative stress can lead to damage to the RBC cytoskeleton, changes in membrane fluidity, and cellular deformability, which can affect the passage of RBC through the microcirculatory network. Reduced erythrocyte deformability was reported in cerebrovascular disorders, acute myocardial infarction, and stroke [134, 141-144].

### 3.3 RBCs deformability in neurodegenerative diseases

Abnormal erythrocyte morphology and functionality have been reported in patients with neurodegenerative diseases. The prevalence of neurodegenerative diseases, particularly Alzheimer’s and Parkinson’s diseases, as well as amyotrophic lateral sclerosis, has significantly increased in the recent decade due to the increase in life expectancy but also due to the lack of early detection and diagnostic procedures [145]. The search for suitable molecular biomarkers is complicated due to interference from plasma proteins and hemolysis. However, erythrocytes avoid the interference problems from blocking proteins (albumin, etc.) encountered during the quantitative analysis of plasma and cerebrospinal fluid. Therefore, cell-based biomarkers such as RBCs could be an appealing option. RBC biomarkers could help differentiate neurodegenerative diseases and assist in earlier diagnostics. The high heterogeneity and poorly defined preclinical stages of patients with neurodegenerative diseases complicate clinical research. This requires cohort studies that would include early stages subjects, continuously collect samples [146], and validate proposed biomarkers that distinguish between different neurodegenerative diseases through prospective cohort studies.

#### 3.3.1 Alzheimer’s disease and RBCs

In the elderly population, AD accounts for approximately 70% of dementia cases. The frequency of AD cases doubles every five years after age 65 [8]. Despite advancements in understanding AD pathophysiology, early and accurate diagnosis of this disease remains challenging [147-148]. Developing biomarkers for early AD detection is crucial for effective treatment [149-151]. Erythrocytes, increasingly recognized as a possible source of biomarkers for AD and other neurodegenerative diseases [152-153], undergo significant changes in morphology in dementia [154-155], potentially playing a crucial role in the early pathogenesis of the disease [1].

Together with fibrinogen, erythrocytes are believed to undergo significant changes in their proteome structure [156] and perform an essential role in AD progression [157-158]. Alterations in erythrocyte membranes fatty acids (FA) composition are associated with cognitive decline and are reported as an early event in the AD pathogenesis trajectory [159-160].

The analysis of erythrocytes in AD has revealed an increase in Young's modulus [9, 154], membrane fluidity [161-163], and erythrocyte deformability [10, 164]. Using AFM and other microscopy approaches, it was possible to demonstrate in AD patients an increased subpopulation of morphologically distinct, elongated erythrocytes with alterations in membrane architecture [158]. In the study by Bester and co-authors [154], substantial changes in RBC morphology and significantly increased membrane stiffness were observed in AD patients with high ferritin levels, suggesting the possibility that iron overload may contribute to an accelerated progression of AD. Abnormal iron metabolism influences alpha-synuclein misfolding and plaque aggregation [165]. The changes in hemorheological properties and cerebral flow are associated with impaired cognitive function in AD patients [166]. These findings underscore the potential of erythrocytes as biomarkers for AD and their role in the disease's progression, providing a clear direction for further research and adding a new dimension to our understanding of AD.

#### 3.3.2 Amyotrophic lateral sclerosis (ALS)

ALS is a progressive, fatal, and mostly sporadic (90-95%) neurodegenerative disease that affects motor neurons of the spinal cord, brainstem, and motor cortex. Recent population-based studies have demonstrated that the age incidence pattern of ALS is similar to AD and Parkinson’s disease (PD) age-dependent neurodegenerative diseases, with a peak of incidence in individuals >80 years old [167]. The deformability of erythrocytes and acetylcholinesterase activity increased in patients with ALS [7]. Moreover, the erythrocyte surface roughness was significantly smoother in ALS patients [11, 168], and ALS patients had higher erythrocyte maximum height, area, and volume and significantly higher membrane stiffness [168]. A larger erythrocyte surface area was found to be an independent predictor of ALS patient's lower survival [11].

#### 3.3.3 Parkinson’s Disease

Parkinson’s disease (PD) is increasing in prevalence and incidence with age, reaching 3% in octogenarians [169]. The erythrocytes of patients with PD and ALS have increased stiffness [9] and are different from healthy subjects in terms of other biophysical parameters [11,152]. It is also known that PD patients exhibit significant morphological changes in erythrocytes, including membrane blebbing, membrane scrambling, and cell shrinkage [158]. These changes are partially mediated by calcium influx [153]. Moreover, the erythrocyte morphological impairment is possibly associated with aggregated alpha-synuclein of erythrocyte membranes [153, 170-172], a potential biomarker for Parkinson’s disease [173-175]. The alpha-synuclein level in healthy subjects’ erythrocytes is approximately 1000 times higher than in cerebrospinal liquid [176-177]. The monomeric and aggregated alpha-synuclein levels significantly increase in the erythrocytes of PD patients compared to healthy individuals [173-174, 178]. A recent study demonstrated a rise in concentrations of Hb complex with alpha-synuclein (Hb^α-Syn^) in human RBCs and brains in an age-dependent manner [179]. The authors hypothesize that since mature RBCs lack most organelles and nuclei, the abundance of alpha-synuclein in RBCs may be explained by its uptake from the plasma and further binding with Hb. Furthermore, alpha-synuclein associated with erythrocyte-derived extracellular vesicles (EVs) can effectively penetrate the BBB via adsorptive-mediated transcytosis and trigger an inflammatory response in microglia [180-181].

Furthermore, the total and oligomeric alpha-synuclein was elevated even in the early motor stage of PD [182], and higher levels of this protein were associated with a faster clinical decline [182]. Vicente Miranda and co-authors [183] reported the amounts of posttranslational modification of alpha-synuclein in erythrocytes of PD patients, including phosphorylation (Y125), nitration (Y39), glycation, and SUMOylation forms. Thus, PD patients have increased levels of glycated forms and reduced levels of SUMOylated alpha-synuclein forms [184]. Various studies have conducted a detailed analysis to determine the biomarker potential of different alpha-synuclein forms (total, oligomeric, post-translationally modified) [185-186].

#### 3.3.4 Multiple system atrophy

Multiple system atrophy (MSA) is a rare, fatal, and rapidly progressive neurodegenerative disease with onset usually in the sixth decade of life [187-188]. Generally, levels of oligomeric and phosphorylated alpha-synuclein are significantly increased in MSA patients. The alpha-synuclein aggregates that are associated with multiple system atrophy and PD correspond to different conformational variants of alpha-synuclein, which can be detected and amplified by alpha-synuclein-Ca^2+^ ATPase [189]. Studies of alpha-synuclein levels in erythrocytes demonstrated increased values of total, oligomeric, and phosphorylated alpha-synuclein in MSA and PD patients compared with controls and decreased values in AD patients [190].

Although most investigations in neurodegenerative diseases are geared toward neurons and glia, erythrocytes are also involved in the pathogenesis of multiple neurodegenerative diseases. The accumulation of alpha-synuclein is an essential step in the development and progression of PD. The EVs derived from RBCs exist in the brain, and alternatively, the EVs derived from the central nervous system exist in the blood and are extensively explored as biomarkers for neurodegenerative diseases [191-194]. Their considerable variability, analysis by different groups of EVs differing by size and origin (exosomes vs. large EVs particles), small cohorts, and lack of independent validation across different groups underscore using EVs as diagnostic biomarkers for neurodegenerative diseases [195].

**Table 1:** Methods for assessing RBC deformability

Method	Principles of the method	Parameter	Advantages	Limitations
**Shear stress ektacytometry**	Applying a series of shear stresses to the cells in a viscous medium resulting in cell elongation (Fig. 3A)	Elongation index. Cellular viscosity (indirect assessment)	Robust and reproducible	Provides only average values for a population of cells
**Osmotic gradient ektacytometry**	Applying a series of osmotic pressures during constant shear stress resulting in cell elongation (Fig. 3A)	50% lysis point Elongation index	Allows osmotic fragility measurement and indirect estimation of S/V, cell surface and cytoplasmic viscosity Robust and reproducible	Provides only average values for a population of cells
**Micropipette aspiration**	Microscopic study of suction of a single RBC into a capillary micropipette (Fig. 3B)	Cell volume and surface area Membrane viscosity Moduli of shear, bending, area, compressibility Relaxation time Young’s modulus	Wide range of available parameters	Low throughput
**RT-DC**	Microfluidic cytometry acquiring images >100,000 cells per experiment in real time (Fig. 4B)	Circularity change Young’s modulus Cell size and volume	Quantitative and qualitative analysis available	Proprietary equipment
**AFM**	High-resolution scanning probe microscopy (Fig. 3C)	Topographic imaging Young’s modulus Relaxation time	Provides a three-dimensional surface profile with very high resolution	Low throughput
**Optical tweezers**	A focused laser beam manipulates the beads attached to the cells (Fig. 3D)	Shear and bending moduli Relaxation time Cell agreggability	Low forces can be applied	Low throughput
**QPI**	Quantifying the phase shift in the light passing through a specimen (Fig. 3E)	Topographic imaging Dynamic membrane fluctuations Cytoplasmic viscosity	Provides an assessment of local membrane properties	Proprietary equipment Low throughput
**Filtration**	Modeling small capillaries of the microvasculature (Fig. 3F)	Flow time, resistance or lysis dynamics	Allows to detect even small subpopulations of the less deformable cells	Provides only average values for a population of cells
**Microsphiltration**	Modeling narrow Inter-endothelial slits	RBCs retention versus flow-through	Allows to detect even small subpopulation of the less deformable cells	Provides only average values for a population of cells
**Microfluidics**	Highly customizable PDMS microchannels featuring micron-scale constrictions paired with microscopes or high-speed cameras (Fig. 5A-F)	Depends on setup Flow time, shear modulus, relaxation time, sustained retention, cell volume and size	Quantitative and qualitative analysis Wide range of scientific applications of different setups	Diversity of chip structure reduces interlaboratory reproducibility

## 4. Methods for assessing deformability of RBCs

**Figure 3.**
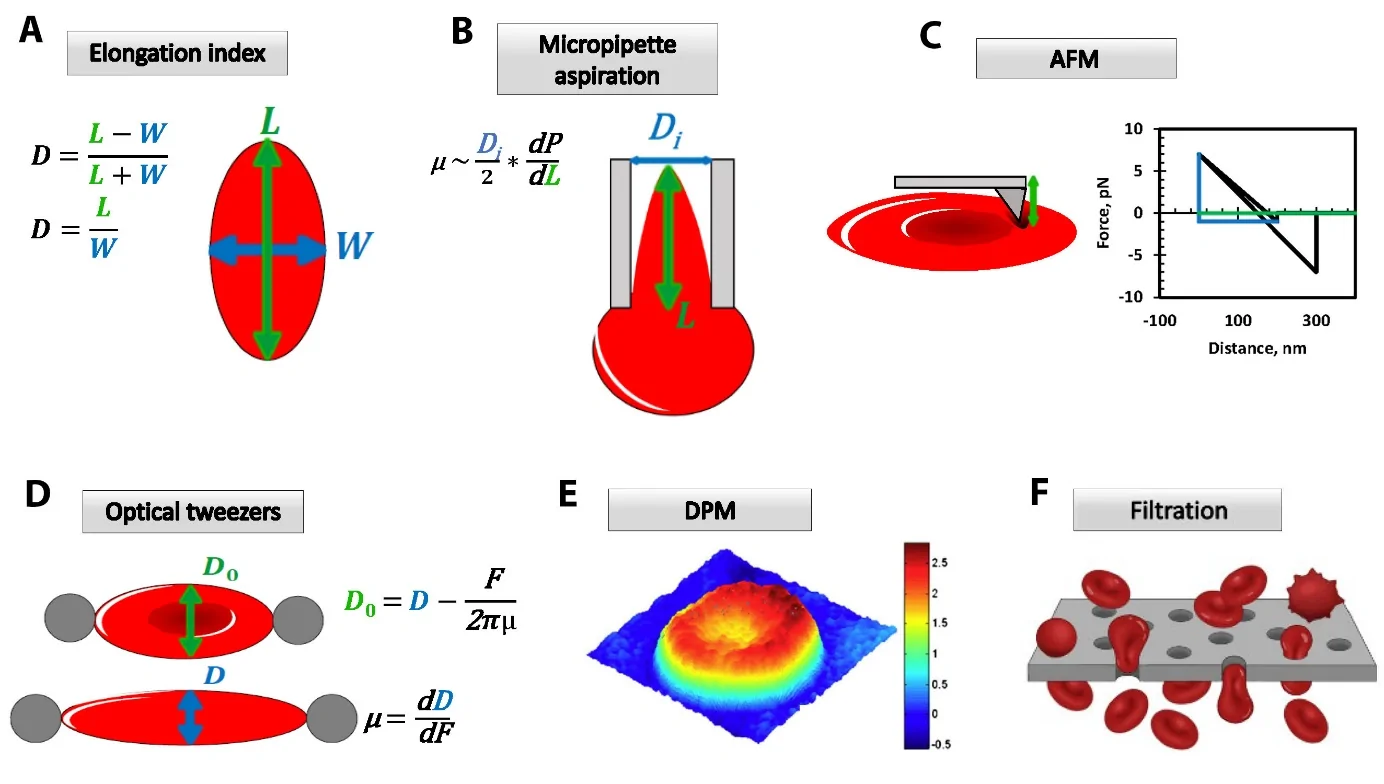
**Different approaches in estimating mechanical properties of RBCs by non-microfluidic methods**. (**A**) Ektacytometry: method provides a diffraction pattern obtained for the cell suspension under different values of applied shear stress in a highly viscous medium; elongation index defined as $EI=\frac{L-W}{L+W}$, where L is the length of the major axis, and W is the length of the minor axis of the diffraction pattern; (**B**) micropipette technique allows to calculate in the preswollen erythrocyte shear modulus defined as $\mu =\frac{\pi D_{i}^{2}}{4}\times \frac{dP}{dL}$, where P is the negative pressure applied to the pipette with the internal diameter Di, and L is the length of the protrusion of the suctioned erythrocyte; (**C**) AFM: a sharp probe is mounted on the end of the cantilever, which is deflected when interacting with the erythrocyte surface. Young’s modulus can be quantified from force versus probe displacement curves; (**D**) optical tweezers: silica or polystyrene microbeads are attached to different sides of an erythrocyte, and after trapping the beads with focused laser beams, a force is applied; a change in the diameter of the erythrocyte allows to calculate shear modulus defined as $\mu =\frac{F}{2\pi \left(D-D_{0}\right)}$, where D_0_ is the initial diameter, and D is the resulting diameter of the erythrocyte; (**E**) diffraction phase microscopy (DPM) - a variant of QPI that utilizes a diffraction grating to construct common-path interferometry combined with fluorescence imaging channel; the DPM image of discocyte is shown, with the color bar representing thickness in microns; (**F**) filtration: this method allows to measure erythrocytes suspension filterability, where F=t_b_/t_s_, and t_s_ and t_b_ are the times of flow through the filter of the same volume of RBC suspension or buffer, respectively.

**Figure 4.**
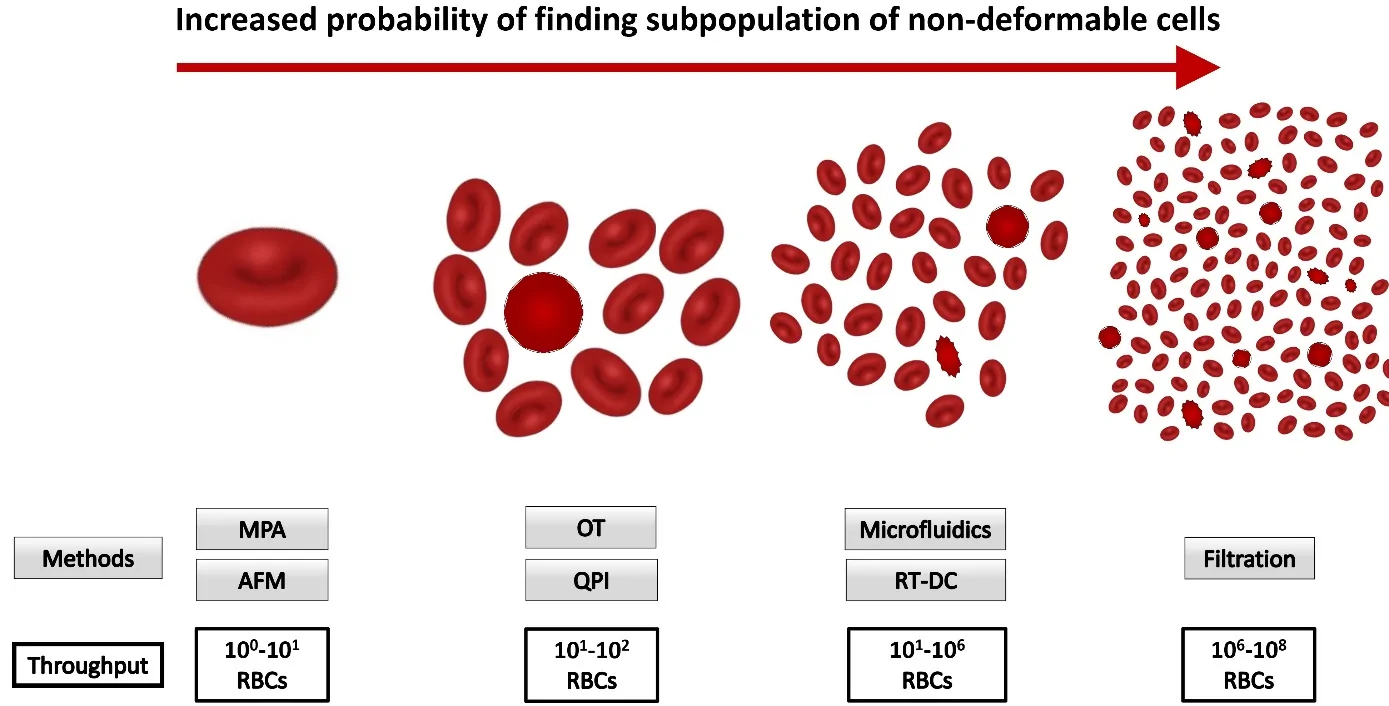
Methods for assessing the mechanical properties of erythrocytes, their throughput and ability to detect subpopulations of non-deformable cells.

There are various methods available for characterizing the biophysical parameters of RBCs [61, 196-200]. They can be divided into two categories: instruments that provide analysis on a population level using whole blood or diluted RBC suspensions and single-cell techniques. A short description of the methods, their advantages and limitations, and measured parameters is presented in Table 1 and Figure 3. The ability of some methods to detect a subpopulation of non-deformed cells is limited by the low throughput (Fig. 4). Bulk flow methods such as ektacytometry are not suitable for subpopulation analysis when a small percentage of the total RBC population has altered deformability. On the contrary, only ektacytometry with its osmotic modification is currently a standardized method with high throughput, making it suitable for clinical settings. While some microfluidic and filtration methods offer high throughput and are relatively easy to manipulate, they lack standardization and commercially available devices.

One of the obligatory conditions for RBCs to pass through narrow capillaries and splenic slits is their ability to elongate. The most widespread measurement for this type of RBC mechanic is the ektacytometer, combining principles of viscosimetry and laser diffractometry to assess shear modulus. The ektacytometer provides a diffraction pattern for the cell suspension under different values of applied shear stress in a highly viscous medium. From the diffraction pattern, the elongation index (EI) is calculated as $\frac{L-W}{L+W}$, where L is the length of the major axis in the direction of the flow, and W is the length of the minor axis perpendicular to the flow (Fig. 4A).

Generally, ektacytometry is viewed as an analysis providing average data for the RBCs population. However, a method for quantifying poorly deformable RBCs has also been proposed [201], albeit not for small cellular subpopulations.

The filtration method is based on the use of filters with pores of 3.5-5 μm in diameter and a length of 10-15 μm. This prevents erythrocytes with reduced deformability from passing through, causing the pores to clog and slowing down the flow. As a result, the method can simulate microcirculation in narrow capillaries and assess microrheology [202-204]. The number of erythrocytes in the suspension can exceed the number of pores by two orders of magnitude, making the method extremely sensitive to the presence of a relatively small population of nonfilterable cells. Unfortunately, this method only provides integral readings [205].

These techniques differ in their capabilities in characterizing small populations of cells with changed deformability (Fig. 4).

### 4.1 Microfluidics-based methods

**Figure 5.**
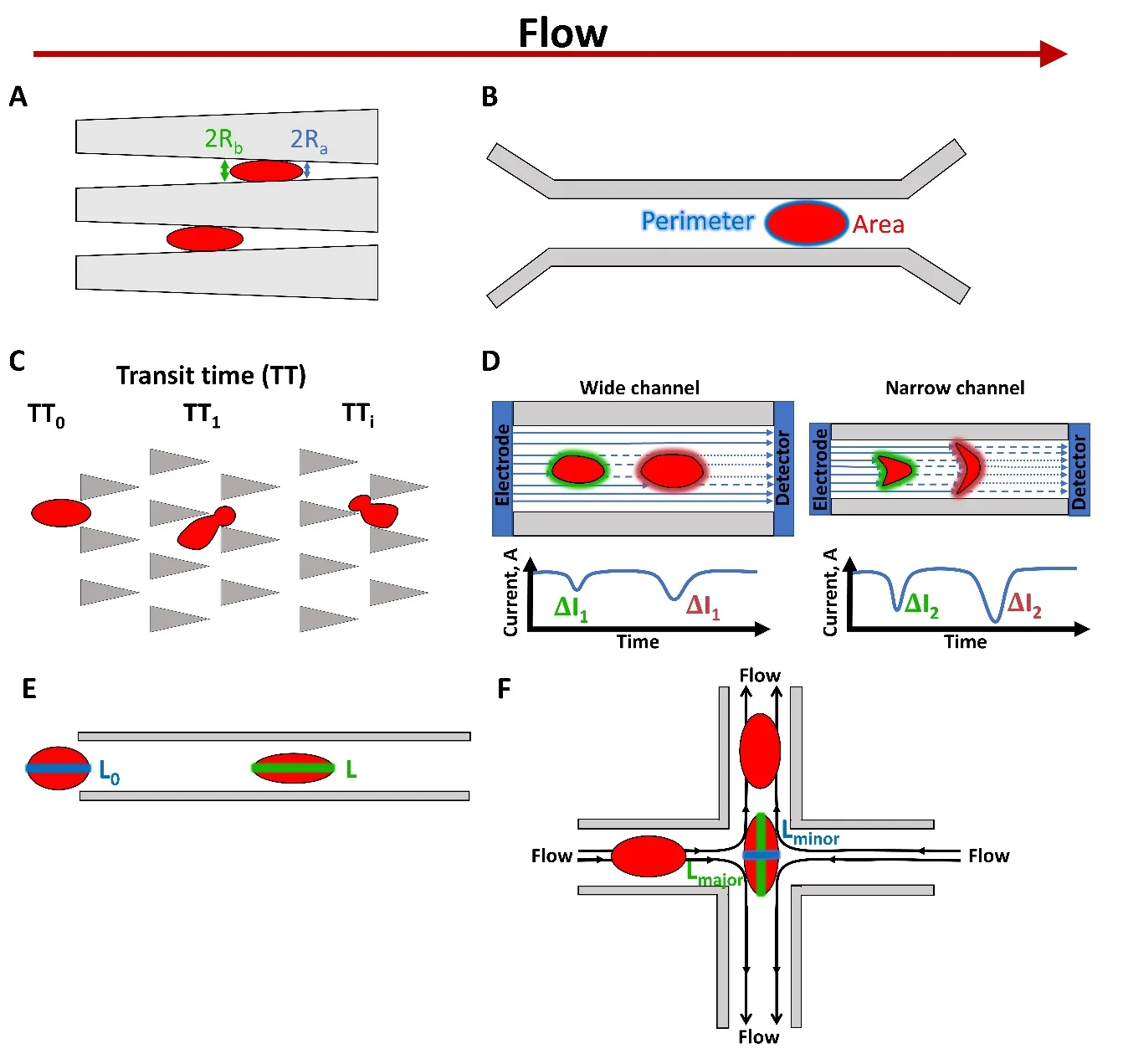
**Selected microfluidic designs to assess the mechanical properties of RBCs**. (**A**) Human erythrocyte microchannel analyzer (HEMA) uses wedge-shaped channels to measure membrane cortical tension, which is defined as $T=\frac{\Delta P}{2\left(\frac{I}{Ra}-\frac{I}{Rb}\right)}$, where ΔP is a pressure drop, Ra is the radius of the anterior part of the erythrocyte and Rb is the posterior end radius; (**B**) RT-DC allows to determine erythrocyte deformation by measuring the cell shape deviation from circularity, defined as $Deformation=1-\frac{2\sqrt{\left(\pi \times \text{ Area }\right)}}{\text{ Perimeter }}$; (**C**) “deformability cytometer’’ combines cytometry with a microfluidic set-up to measure cell transit time and calculated velocity, providing a representation of the deformability of the erythrocytes; (**D**) a microfluidic set-up based on electrical measurements of erythrocytes involves the flow of erythrocytes in channels with different cross-sectional areas; changes in the current are detected depending on size, volume, deformability of the erythrocytes; (**E and F**) microfluidic approaches that allow measurement of erythrocyte elongation parameters: the ratio of major axis length before and after the erythrocyte enters the constriction $EI=\frac{L}{L_{0}}$, (**F**), and index $DI=\frac{L_{major\;}}{L_{minor}}$, where L_major_ and L_minor_ are the lengths of major and minor axes, respectively.

The microfluidic technique has many setup options and devices have individual geometry in each laboratory. Instruments vary not only the geometry of the channel construction but also in the design of the setup and parameters measured (Fig. 5A-C).

Microfluidics readouts for deformability include deformation index, RBC transit time/velocity, pressure threshold [206], Young’s modulus [207], electric impedance [208], channel clogging [209], cell margination [210], and lysis rate. Importantly, many microfluidics techniques, such as real-time deformability cytometry (RT-DC) [211-212] and biophysical cytometry [213-214], allow for single-cell analysis of erythrocyte deformability. An interesting microfluidic approach mimicking *in vitro* splenic filtration was described by Qiang and co-authors [215].

A functional assay complementary to ektacytometry and capable of detecting small-fraction RBCs with abnormal deformability was developed using the OcclusionChip technology, which mimicks the capillary bed architecture [216-219]. The OcclusionChip assay is used to calculate the occlusion index, which some researchers suggested as a biomarker for cell deformability [220-221]. For a more comprehensive review of microfluidics for assessing the physical properties of RBCs, see reviews [222-224].

## 5. Single cell analysis of RBCs

The geometry and shape of the RBC, including the ratio of cell surface and cell volume, is another essential erythrocyte characteristic that correlates with deformability and can be analyzed using ellipticity, extent, eccentricity, cellular circularity, and solidity parameters. An increase in the cell volume (i.e., mean corpuscular volume, MCV) has been reported in elderly healthy subjects [225-228]. MCV of erythrocytes is a hematological biomarker that provides information at the population level, and the high heterogeneity of RBC may require statistically robust single-cell analysis methods. An increase in MCV has been linked to lower global mental status, even after adjusting for potential confounders [229], and lowered reaction time [230]. Furthermore, in the Chinese Health and Retirement Longitudinal Study, it was found that higher MCV (>100 fL) was significantly associated with decreased global cognitive function, episodic memory, and mental status, but this association was found to be significant only in male subjects [231].

### 5.1 RDW as a parameter of RBCs heterogeneity

Red blood cell distribution width (RDW) is an index of size heterogeneity (anisocytosis) of the RBCs, calculated with the following formula: (statistical deviation (SD) of RBCs’ volumes)/ MCV) × 100%. Elevated RDW values are linked to inflammatory markers in numerous diseases. RDW values above the normal range indicate the presence of small (microcytes), large (macrocytes), or both cell types in blood and are associated with decreased deformability [232-233] and elongation index [234]. The senescence of erythrocytes contributes to the heterogeneity of erythrocyte populations, resulting in cell type-specific alterations that differ from other cell types where changes in DNA-methylation patterns are important [235]. The heterogeneity of RBCs, i.e., RDW values, increases progressively with age, with a stronger correlation in women [236-240]. High RDW levels are associated with reduced survival, high morbidity in elderly patients after surgeries [241-243], adverse long-term outcomes, higher all-cause mortality in elderly with hip fractures [244], frailty [245], increased mortality in critically ill nonagenarians in the intensive unit [246].

Elevated RDW has been identified as an independent risk factor for cognitive dysfunction and severe forms of Alzheimer’s disease [86, 89, 230, 247]. The exact mechanisms underlying the association of increased RDW with dementia are not fully understood and stay speculative. However, high RDW levels may serve as a predictive indicator for hypoxia and cerebrovascular problems and trigger metabolic changes due to decreased deformability of RBC [232, 248-249]. Furthermore, RDW levels are found to be significantly higher in PD patients compared to normal individuals [250-252], but there was no relation found between the severity of PD, duration of disease, and RDW levels [251].

### 5.2 RBCs aggregation

Blood and RBC aggregate properties can change due to various factors, such as changes in RBC age, blood plasma composition, temperature, and the clinical patient state [253-256]. Erythrocytes with small shear forces can form two-dimensional (rouleau) and three-dimensional aggregates, which disaggregate under increasing shear stresses. Methods used to analyze aggregation of RBCs are focused on analyzing erythrocyte aggregation at the population level, providing averaged values and leaving behind small subpopulations of platelet-erythrocyte aggregates and erythrocyte clusters with myeloid cells.

Moreover, the aggregation of RBCs depends on electrostatic repulsion and cell deformability. It is generally thought that decreased deformability is associated with increased aggregability, and the integral membrane protein band 3 is part of membrane-centered machinery that plays a crucial role in both processes—aggregation and deformability [257]. Due to membrane reorganization, senescent erythrocytes have increased adhesion to endothelial cells [258]. A decreased erythrocyte deformability is associated with a heightened thrombotic potential, as more rigid erythrocytes can easily occlude micro-vessels, disrupt blood flow, and affect platelet activation [259].

The erythrocytes of older donors demonstrated a significant decrease in membrane sialylation [260]. It was shown that the removal of sialic acid from the erythrocyte surface induces abnormal erythrocyte adhesion [261]. Sialic acid content is essential for maintaining the negative surface charge of RBC and influences erythrocyte deformability and rheological parameters [262-263].

In humans, 0.1-0.3% of circulating RBCs are part of platelet-RBC clusters [264]. Recently, a novel mechanism for RBC clearance involving the formation of platelet RBC complexes (P-RBCs) was proposed [264]. The authors advanced our understanding of canonical erythrophagocytosis when RBCs with decreased deformability are lysed and cleared by the reticuloendothelial system and splenic macrophages [265]. The platelet-dependent clearance was described using an imaging flow cytometry approach, which allows detailed analysis of cellular clusters [266-267].

Recent studies have also provided evidence for brain endothelial erythrophagocytosis of oxidatively stressed or senescent RBC with reduced deformability by murine and human brain endothelial cells [268-270]. Increased RBC interactions with the endothelium of brain capillaries may represent an alternate source of cerebral microhemorrhage development distinct from the traditional RBC extravasation to the brain parenchyma. This finding may have clinical implications for conditions chracterized by increased RBC stress (such as aging RBCs) [270].

### 5.3 Imaging Flow Cytometry in characterization of heterogenous RBCs

**Figure 6.**
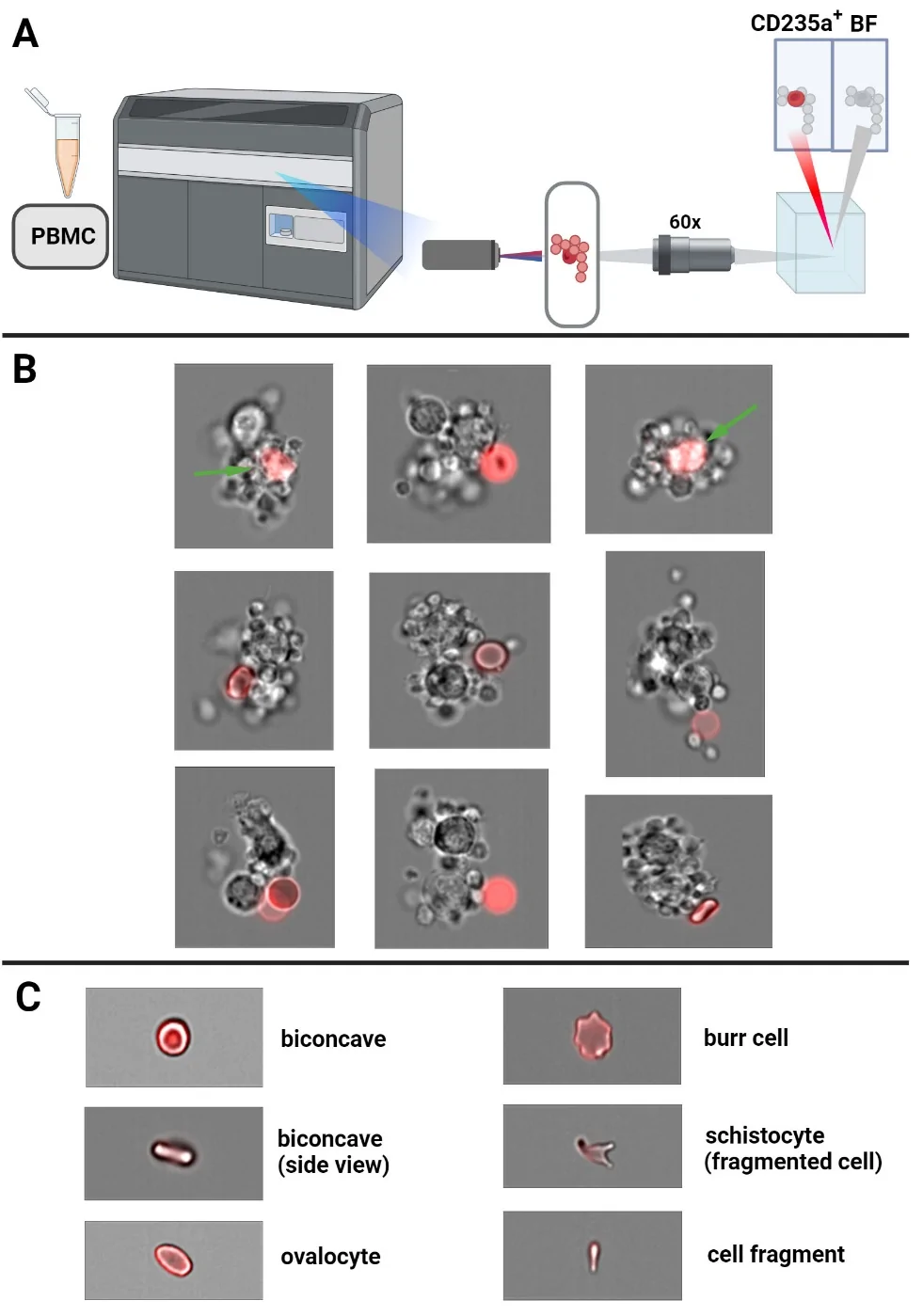
**Imaging flow cytometry analysis of PBMCs aggregates**. (**A**) Schematic representation of the Imagestream X Mark II (IsX) multispectral imaging flow cytometer (Created with Biorender); (**B**) Gallery of cellular aggregates stained with antibodies to glycophorin (CD235a^+^); C. Selected RBCs images. Acquired with IsX (Amnis-Cytek, USA; Lasers: 488 nm, 637 nm; objective: 60x). Green arrows denote representative stained RBCs.

The development of conventional flow cytometry and fluorescent microscopy has led to imaging flow cytometry (IFC), a hybrid technique that combines the best features of the two methods [271-272]. IFC allows for the high-throughput analysis of highly heterogeneous cellular populations, providing an advantage for studying heterogeneous RBCs, cell clusters, red-cell derived particles [273], and small populations of erythroid progenitors present in the blood (Fig. 6). Cellular populations, such as RBCs, can be analysed for heterogeneity using a combination of FACS-based sorting and following single-cell techniques [274-275]. Until recently, cell sorting methods were based mainly on conventional flow cytometry. Recent developments include image-based sorting [276] and spectral-based cytometry sorters (FP7000, SONY Biotechnology Inc.), which are still left for future cell sorting that would utilize biophysical parameters such as cell deformability.

In the last decade, IFC has been used for assessing the quality of stored RBCs [277-279], analysis of RBCs in sickle cell anemia [273, 280-282] and malaria [283-284], characterizing hemoglobin distribution [285], and studying RBC aggregation [286]. Changes in erythrocyte shape depend on the mechanical deformability of the cellular membrane and cytoplasm. Cells from older donors display progressively more irregularities in their shape [287], and IFC may provide high-throughput phenotyping of RBCs, which could serve as a proxy for shifts in erythrocyte deformability. The potential of IFC analysis in RBC characterization can be further enhanced by using machine learning and artificial intelligence algorithm approaches [288-289], allowing for the analysis of RBCs and their aggregates (Fig. 6).

The analysis of large patient-oriented cohorts is required to decipher the fundamental association between RBCs’ shape, deformability, and receiving a biophysical signature of erythrocyte aging. Implementation of deep learning in microscopy demonstrated the ability to predict the deformability measured by a microfluidic cell sorting device [290]. Utilizing IFC in conjunction with methods for deformability estimation provides ample opportunities for future research/

## 6. Conclusion and future perspectives

As human life expectancy increases globally, there is a rapid rise in neurodegenerative diseases and chronic inflammatory pathologies associated with aging. Therefore, it is crucial to find reliable biomarkers that can predict the risk of age-related pathologies. The biophysical markers, which can integrate multiple cellular parameters and signal transduction pathways, may provide a more global picture of disease progression and outcome. Some studies show that the measure of single biophysical parameters, such as erythrocyte deformability or a combination of biophysical parameters, could serve as an indicator of disease severity and a predictive indicator of the disease outcome. We postulate that in tandem with highly sensitive methods for assessing deformability, it will be possible to analyse and study erythrocyte subpopulations that may be of clinical relevance to aging and related diseases. This approach has several potential applications, such as: (1) preclinical screening of therapeutics in elderly populations; (2) early diagnostics of neurodegenerative diseases and predicting adverse outcomes in elderly patients; (3) using erythrocyte-based biomarkers to help clinicians determine whether a patient is a candidate for surgery; (4) evaluating various age-related diseases and frailty to potentially predict a trajectory of disease progression; and (5) to measure the efficacy of the drugs. The recent findings that Yoda1, a small molecular that activates the mechanosensitive ion channel protein PIEZO1, has beneficial effects on aging mice and boosts the effect of PIEZO1 on lymphatic vessels, suggest potential therapeutic effects of PIEZO1 agonists not only on mLVs but also on erythrocyte deformability. Consequentially, this could have a positive effect on brain functionality in aging patients and neurodegenerative diseases prevention and therapy. We anticipate that affordable and accessible biophysical biomarkers could serve as complementary and predictive metrics for screening elderly patients in early asymptomatic stages of neurodegenerative diseases, helping to identify at-risk patients quickly.
